# Convergence between Development and Stress: Ectopic Xylem Formation in Arabidopsis Hypocotyl in Response to 24-Epibrassinolide and Cadmium

**DOI:** 10.3390/plants11233278

**Published:** 2022-11-28

**Authors:** Diego Piacentini, Federica Della Rovere, Simone D’Angeli, Laura Fattorini, Giuseppina Falasca, Camilla Betti, Maria Maddalena Altamura

**Affiliations:** 1 Department of Environmental Biology, Sapienza University of Rome, 00185 Rome, Italy; 2 Department of Biosciences, University of Milan, 20133 Milan, Italy

**Keywords:** brassinosteroids, Cadmium, ectopic vascular differentiation, hypocotyl pericycle, xylary identity

## Abstract

Ectopic xylary element (EXE) formation in planta is a poorly investigated process, and it is unknown if it occurs as a response to the soil pollutant Cadmium (Cd). The pericycle cells of *Arabidopsis thaliana* hypocotyl give rise to EXEs under specific hormonal inputs. Cadmium triggers pericycle responses, but its role in EXE formation is unknown. Brassinosteroids (BRs) affect numerous developmental events, including xylogenesis in vitro, and their exogenous application by 24-epibrassinolide (eBL) helps to alleviate Cd-stress by increasing lateral/adventitious rooting. Epibrassinolide’s effects on EXEs in planta are unknown, as well as its relationship with Cd in the control of the process. The research aims to establish an eBL role in pericycle EXE formation, a Cd role in the same process, and the possible interaction between the two. Results show that 1 nM eBL causes an identity reversal between the metaxylem and protoxylem within the stele, and its combination with Cd reduces the event. All eBL concentrations increase EXEs, also affecting xylary identity by changing from protoxylem to metaxylem in a concentration-dependent manner. Cadmium does not affect EXE identity but increases EXEs when combined with eBL. The results suggest that eBL produces EXEs to form a mechanical barrier against the pollutant.

## 1. Introduction

The pericycle of the hypocotyl is contiguous to the primary root pericycle and is the most external layer of the hypocotyl primary vascular system (stele). In many plants, including Arabidopsis, the stele shows exarch protoxylem and endarch metaxylem [1]. The pericycle consists of cells able to change their fate during the primary growth by asymmetric cell divisions [2], giving rise to adventitious root primordia by anticlinal cell divisions [3] and to ectopic xylary cells (EXEs) by periclinal cell divisions, respectively [4,5]. Moreover, during secondary vascular growth, the pericycle cells transdifferentiate into vascular cambium [1,2]. During primary growth, auxins (indole 3-acetic acid and its precursor indole-3-butyric acid) are known to control pericycle proliferation, leading to either adventitious rooting or EXE formation [3,6]. However, different from the widely investigated role in adventitious rooting, their involvement in the process of ectopic xylem formation still needs investigation.

The EXE formation has been described in various in vitro culture systems by using histological and epifluorescence techniques, e.g., in *Zinnia elegans* single mesophyll cells [7], in tobacco and Arabidopsis thin cell layers [4,8], in Arabidopsis leaf discs, but more recently also by the use of the Vascular Induction System Using Arabidopsis Leaves (VISUAL) [9,10]. The process is known as xylogenesis in vitro and can occur either through direct transdifferentiation of the cells in culture [11,12] or indirectly by a cell proliferation phase followed by de novo EXE differentiation [4].

The entire plant or seedling is less responsive to changes in developmental programs than single cells or organ explants cultured in vitro because the compensative events that occur in the entire organism preserve its normal ontogenesis. For this reason, the EXE formation, known as xylogenesis in planta, has been less studied than xylogenesis in vitro. The limited information available in the literature describes xylogenesis in planta as an indirect process with a phase of cell proliferation preceding the de novo EXEs differentiation, as reported for the hypocotyls of Arabidopsis seedlings [4]. In in vitro xylogenesis, the response of the excised single cells/cell systems is induced by wounding, which alters the hormonal balance of auxin [13]. By contrast, the important characteristic of EXE formation in planta is that it occurs in intact organs, in which EXE formation is not a consequence of a wounding reaction [14]. Still, it is one of the manifestations of the plant’s compensatory/repair responses to the action of external disturbing agents.

Genes in common between xylem formation in the stele and xylogenesis in vitro and between xylem formation in the stele and EXE formation in planta have been found [4,15], as well as transcriptional switches of protoxylem vs. metaxylem [16]. This suggests that the combined information about xylem development in the stele in planta and xylogenesis in vitro might be useful for understanding factors involved in EXE formation in planta despite the diversity of the processes.

A good system for studying EXE formation in planta is the basal hypocotyl of seedlings of the model plant *Arabidopsis thaliana* [4]. In Arabidopsis seedlings, the EXEs exhibit either helical/annular or pitted/densely reticulate thickenings, the same as the protoxylem and the metaxylem, respectively, of the hypocotyl stele [5]. Moreover, the EXEs formation, and their xylary identity (either protoxylem or metaxylem), are affected by different auxins, with the cooperation of ethylene and jasmonates, but also by other molecules, including some transcription factors [4,5,17,18].

Brassinosteroids (BRs) are polyhydroxylated steroids that affect numerous plant developmental processes, including the differentiation of primary and secondary vascular cells in planta [19] and of EXEs in xylogenesis in vitro [10,20]. Moreover, the vasculature abnormalities occurring in BR-deficient mutants [21,22], together with the positive effects of exogenous BRs applied as 24-epibrassinolide (eBL) at nanomolar (nM) concentrations on direct/indirect xylogenesis in vitro [10,23], clearly suggest the involvement of these hormones for the EXE process in planta, and probably for the definition of the xylary identity [4,7,18,24].

The perception of BR hormones starts at the plasma membrane, where they bind to the leucine-rich-receptors (LRRs) BRASSINOSTEROID INSENSITIVE1 (BRI1) and its two paralogs. Ligand binding to BRI1 leads to kinase domains activation that causes a cascade of intracellular phosphorylation events that end with the transcription factors BRI1-EMS SUPPRESSOR1 (BES1/BZR2) and BRASSINAZOLE-RESISTANT1 (BZR1), which drive the expression of BR-related genes affecting specific developmental programs [25]. BES1 has been reported to promote xylem differentiation [26,27], while exogenous BRs exert positive effects on xylary cell formation, with eBL as one of the most bioactive BRs [28]. Recently, it has been demonstrated that, in comparison with the BR-untreated seedlings, the secondary vascular system of *Pinus massoniana* seedlings treated with exogenous BRs shows xylem thickness and improved number of xylem cell layers which increase together with the increasing of BR concentration [29]. Taken together, there is a consistent possibility that eBL, applied to Arabidopsis seedlings in a range of low concentrations, might cause a significant promotion of pericycle periclinal proliferation and EXE formation. Moreover, BRs are known as multidimensional regulators of plant development and stress responses [30] because their exogenous application helps to alleviate numerous biotic and abiotic stresses in plants [31]. For example, their application increases tolerance to Cadmium (Cd), e.g., in tomato [32], rice [33], and Arabidopsis [25]. In the latter, it has been recently demonstrated that exogenous eBL nullifies the negative effects of Cd on the apical structure of the primary root and strongly increases lateral and adventitious root formation [34].

Although these studies provide insight into the BR role(s) in the coordination of plant development and stress response, information about the relationship between BRs and Cd in the control of EXE formation in planta is still lacking. We think that this relationship is possible because, in barley roots, Cd stimulates premature xylem formation within the stele [35]. Moreover, the pericycle is a tissue targeted by Cd, which affects the responses of its cells, e.g., by increasing cell wall thickening, as in durum wheat [36], or by enhancing their number, as in bean roots [37].

The present research aims to establish the role of the exogenously applied eBL in EXE formation from the pericycle and EXE identity, the Cd role in the same processes, and the effects coming from the combination of the phytohormone with the pollutant. The goal is to understand whether this BR and Cd affect EXE formation and whether their combination enhances the ectopic response, with a consequent improvement of the seedling’s mechanical strength in response to the pollutant.

Understanding the effects of still under-investigated hormones, such as BRs, and of a pollutant (Cd), whose morphogenic effects are still partially known, on the same and still little-known developmental process, such as ectopic xylem formation, will contribute to shedding light on the mechanisms that coordinate growth events and stress responses, which are crucial for engineering crops with optimized responses to the pollutant.

## 2. Results

### 2.1. Exogenous 24-Epibrassinolide at 1 nM Causes Significant Reversal of Metaxylem to Protoxylem Inside the Hypocotyl Stele

**Figure 1:**
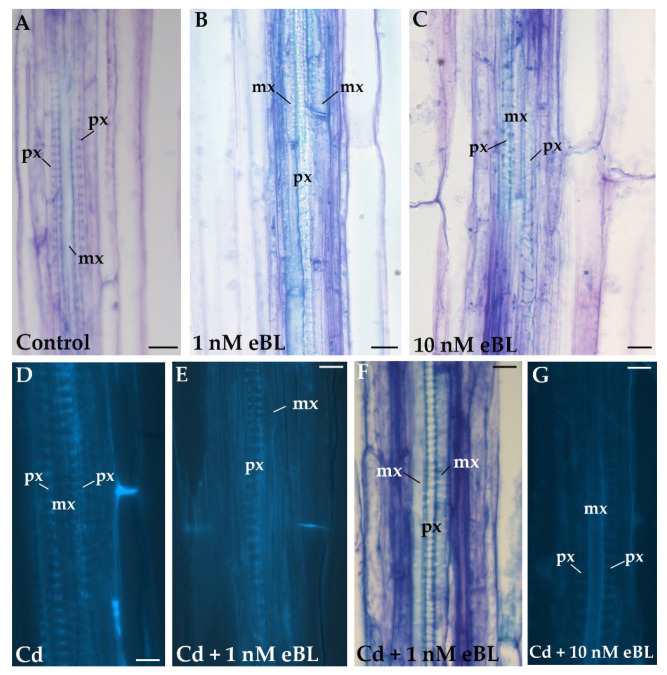
Stelar structure of the basal hypocotyl of *A. thaliana* (Col ecotype) seedlings cultured for 9 days under continuous darkness followed by 7 days under 16 h light/8 h darkness photoperiod with different treatments. Stelar structure with endarch metaxylem and exarch protoxylem in the control (**A**), in seedlings cultured with 10 nM of 24-epibrassinolide (eBL) (**C**), 60 μM CdSO_4_ (Cd) (**D**), 60 μM Cd + 10 nM eBL (**G**). The reversal in stelar structure showing exarch metaxylem and endarch protoxylem in seedlings treated with 1 nM eBL (**B**) and 60 μM Cd + 1 nM eBL (**E**,**F**). Light microscopy images of radial longitudinal sections stained with toluidine blue (**A**–**C**,**F**) or visualized in fluorescence to detect lignin (bright blue color) (**D**,**E**,**G**). mx, metaxylem; px, protoxylem. Bars = 10 µm.

The stelar structure of the basal hypocotyl was histologically investigated to reveal possible anomalies caused by the treatments. No case of xylary identity change occurred in the stele of the untreated controls because the same as in the stele of the contiguous primary root, the metaxylem was endarch, i.e., positioned in the center of the stele, and the protoxylem was exarch, i.e., at the flanks of metaxylem (Figure 1A). The treatment with 1 nM of 24-epibrassinolide (eBL) caused a significant (43%) percentage of seedlings showing a positioning reversal between protoxylem and metaxylem, with endarch protoxylem and exarch metaxylem (Figure 1B). The same phenomenon occurred in 10% of the 10 nM eBL-treated seedlings, although most of the seedlings showed no reversal of xylary identity in this treatment (Figure 1C), as the total ones of the 1 µM eBL-treatment, collectively revealing that the reversal phenomenon was inversely related to the applied concentration.

When the seedlings were treated with Cadmium (Cd) alone, no reversal in position between the protoxylem and metaxylem was observed in the stele (Figure 1D), and the same occurred when Cd was combined with 10 nM eBL (Figure 1G). By contrast, a low but significant percentage (12.5%) of the seedlings treated with Cd + 1 nM eBL showed the reversal (Figure 1E,F).

Taken together, the results show that the change in xylary identity within the stele is specifically related to eBL and its concentrations, whereas the pollutant does not cause the event but counteracts it at the most inductive eBL concentration (1 nM), even if it is unable to nullify it.

### 2.2. Exogenous 24-Epibrassinolide Enhances the Hypocotyl Pericycle Periclinal Proliferation but Not in Cd Presence

**Figure 2:**
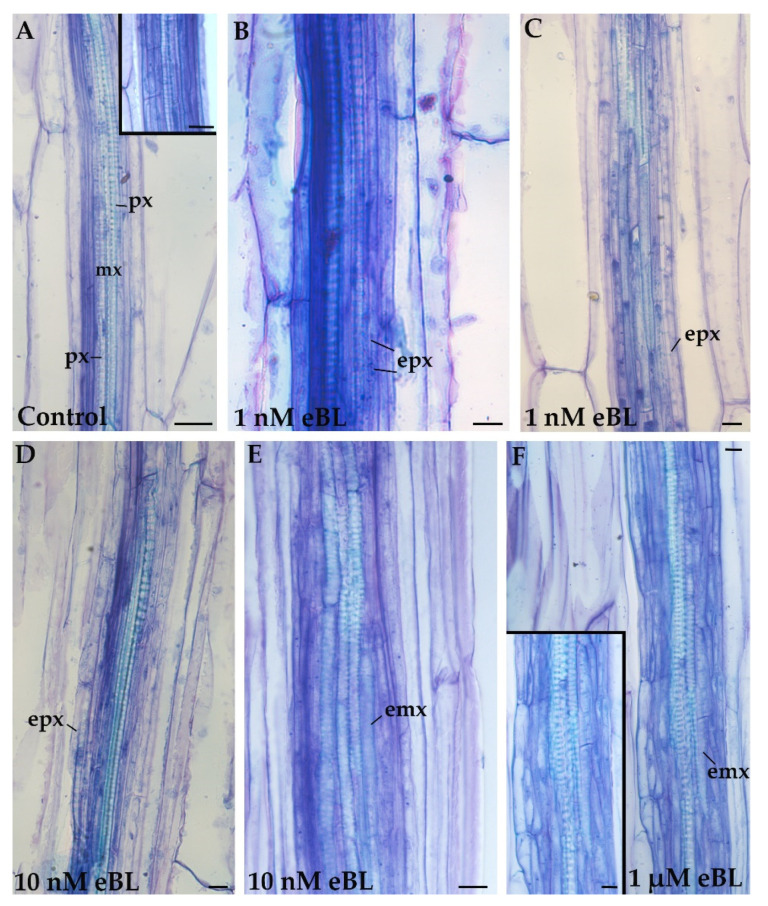
Xylogenesis in the basal hypocotyl of *A. thaliana* (Col ecotype) seedlings cultured for 9 days under continuous darkness followed by 7 days under 16 h light/8 h darkness photoperiod with different treatments. Reduced pericycle cell proliferation in the Control (**A** and *Inset*). Ectopic protoxylem (epx) elements formed in the presence of 1 nM of 24-epibrassinolide (eBL) (**B**,**C**). Ectopic protoxylem (epx) and ectopic metaxylem (emx) elements formed in the presence of 10 nM eBL (**D**,**E**). Ectopic metaxylem (emx) elements formed in the presence of 1 nM eBL (**F** and *Inset*). emx, ectopic metaxylem; epx, ectopic protoxylem; mx, metaxylem; px, protoxylem. Radial longitudinal sections stained with toluidine blue. Bars = 10 µm.

**Figure 3:**
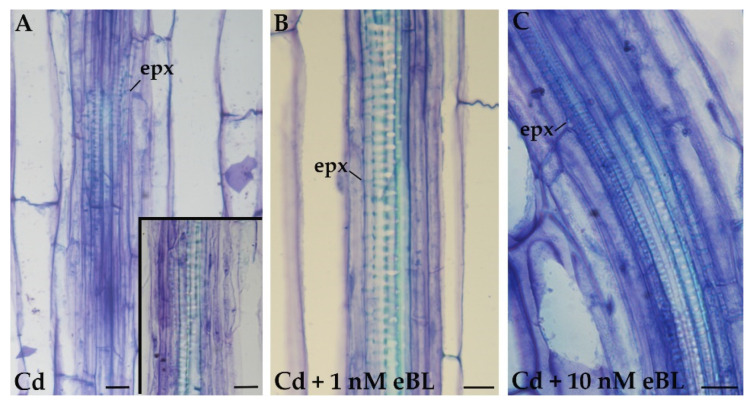
Xylogenesis in the basal hypocotyl of *A. thaliana* (Col ecotype) seedlings cultured for 9 days under continuous darkness followed by 7 days under 16 h light/8 h darkness photoperiod with different treatments. Ectopic protoxylem (epx) elements formed in the 60 μM CdSO_4_ (Cd) treatment (**A**), and reduced pericycle cell proliferation in the same treatment (*Inset*). (**B**,**C**) Production of only epx elements with 60 μM Cd + 1 nM of 24-epibrassinolide (eBL) (**B**) or with 60 μM Cd + 10 nM eBL (**C**). epx; ectopic protoxylem. Radial longitudinal sections stained with toluidine blue. Bars = 10 µm.

At the end of the culture period, the basal portion of the hypocotyl was radially measured, including the stelar region, the pericycle, and its derivatives, when present. The histological analysis revealed that in no case did the pericycle cells transdifferentiate directly into EXEs and that the pericycle derivative cells were always de novo formed by periclinal division (Figure 2 and Figure 3).

**Figure 4:**
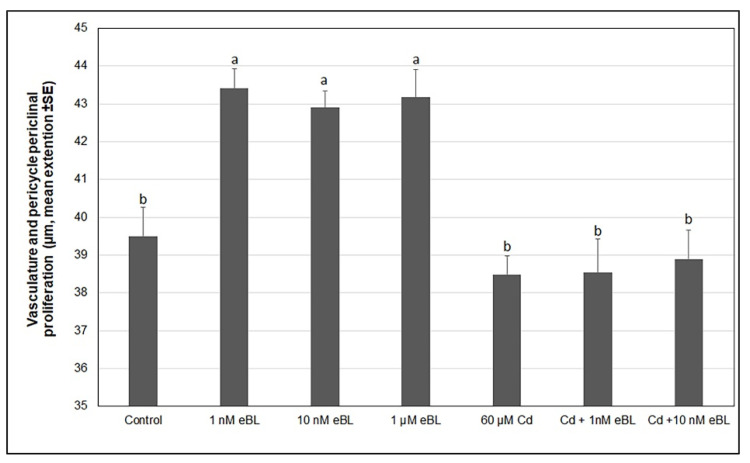
Pericycle periclinal proliferation in the basal hypocotyl of *A. thaliana* (Col ecotype) seedlings cultured for 9 days under continuous darkness followed by 7 days under 16 h light/8 h darkness photoperiod on 1/2 MS medium (control treatment) or on the same medium added with either 1 nM of 24-epibrassinolide (eBL), or 10 nM eBL, or 1 µM eBL, or 60 µM CdSO_4_ (Cd), or Cd + 1 nM eBL or Cd + 10 nM eBL. Letters show significant differences for at least *p* < 0.01. The same letter shows no significant difference. N = 30. Data from the first replicate.

No pericycle cell proliferation, or very reduced, occurred both in the controls (untreated seedlings) and in Cd-alone-treated seedlings (Figure 2A and Figure 3A, and Insets), without significant differences between the two treatments (Figure 4). The combination of Cd with the highest eBL concentration (1 µM) resulted in a very stunted seedling growth (Figure S1), confirming previous results [34]. For this reason, the histological analysis was carried out on the seedlings treated with Cd combined with 1 nM or 10 nM eBL and not with eBL at 1 µM concentration.

No significant increase in the extension of the pericycle proliferation occurred in seedlings treated with Cd combined with either 1 nM or 10 nM eBL compared to the control and the Cd alone treatments (Figure 4). Differently, single treatments with each of the three eBL concentrations caused a conspicuous periclinal proliferation of the pericycle (Figure 4), which was significantly higher than that detected in the control and in the Cd (±eBL) treated seedlings. However, there was no significant difference among the three eBL treatments (Figure 4).

### 2.3. Exogenous 24-EpiBrassinolide Affects Ectopic Xylary Identity in a Concentration-Dependent Manner

**Figure 5:**
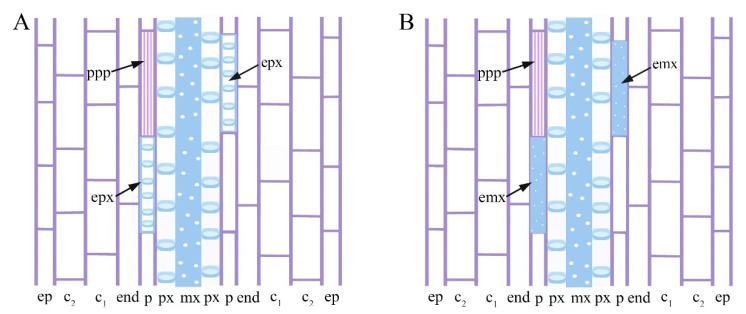
(**A**,**B**) Scheme showing the radial longitudinal section of the basal portion of the hypocotyl of a seedling of Arabidopsis thaliana (Col ecotype) cultured for 9 days under continuous darkness followed by 7 days under 16 h light/8 h darkness with either 1 nM 24-epibrassinolide (eBL) ± 60 µM of CdSO_4_ (Cd) (**A**) or with 1 µM eBL (**B**). Note that the ectopic protoxylem elements (epx, arrows in **A**) and the ectopic metaxylem elements (emx, arrows in **B**) are scattered in the pericycle periclinal proliferated (PPP, arrows) cells. The tissues external to the primary xylem of the vasculature are indicated by the abbreviations: ep, epidermis; c_1_ and c_2_, cortical layers; end, endodermis; p, pericycle.

The pericycle showed proliferation not followed by ectopic xylary elements (EXE) formation (Figure 2A and Inset), or proliferation followed by the formation of ectopic protoxylem (Figure 2B–D and Figure 3A–C), or proliferation followed by the formation of ectopic metaxylem (Figure 2E,F), as schematized in Figure 5. The proliferation without EXE formation occurred not only in the control seedlings (Figure 2A and Inset) and in the Cd alone treated ones (Figure 3A Inset) but occasionally in every other treatment.

The production of EXEs was quantified according to the procedure described in Materials and Methods. All the eBL single treatments produced significantly more EXEs than the untreated controls (Table 1). However, there was an evident concentration-dependent effect on the EXE identity (Table 1) because the ectopic protoxylem elements were prevalently differentiated at 1 nM eBL (Figure 2B,C, Table 1), and both ectopic protoxylem and ectopic metaxylem were produced with 10 nM eBL (Figure 2D,E, Table 1). In contrast, the metaxylem elements were the preferentially produced EXEs at 1 µM eBL (Figure 2F, Table 1). Collectively, the data show a positive role for eBL in EXE formation and xylary identity.

### 2.4. Cd Produces Ectopic Protoxylem, and the Addition of 24-Epibrassinolide Does Not Change the Response

The production of EXEs was also quantified in the treatments with Cd alone or Cd combined with eBL. No significant change in the production of EXEs occurred when Cd alone was compared to the untreated controls (Table 1). In contrast, the combination of Cd with each of the two selected eBL concentrations significantly changed this result (Table 1). In fact, the EXE production under Cd combined with either 1 nM or 10 nM eBL was enhanced, reaching mean values comparable, even if lower, than those of the three treatments with eBL alone (Table 1).

Regarding XE identity, ectopic protoxylem elements prevalently appeared in the poor pericycle proliferation formed under the Cd alone treatment, and the same occurred in the controls (Figure 3A and Table 1). The EXEs prevalently showed protoxylem identity in the two Cd+eBL treatments, without significant differences between the two and with the Cd alone and Control treatments (Figure 3B,C and Table 1).

Altogether, the results show that the addition of BRs to Cd cannot change the ectopic xylary identity, but it can increase the EXE productivity.

## 3. Discussion

Results show that 24-epibrassinolide (eBL) positively affects hypocotyl pericycle proliferation, ectopic xylary element (EXE) formation, and xylary identity. Cadmium (Cd) does not affect xylary identity but increases EXE formation when combined with eBL, suggesting that the latter compound antagonizes its effects through the morphogenic process of ectopic xylary formation in the pericycle derivatives. The study provides new insights into the coordination of plant growth and Cd-stress response, examining events occurring in a specific tissue/developmental context.

Despite being embryonic in origin, the hypocotyl is a plastic organ, able to acquire self-organizing properties during post-embryonic growth to allow adaptability to an unfriendly environment, e.g., that caused by Cd pollution [38]. Present data show that the formation of ectopic protoxylem and metaxylem occur in the basal hypocotyl as an indirect process starting from the pericycle periclinal derivatives, with this occurring under specific eBL treatments, with and without Cd. Results show that the pericycle cells are engaged in two morphogenic events, periclinal proliferation and EXE differentiation, with the first not necessarily followed by the second. The periclinal divisions are known to be parallel to the organ surface and to cause an increase in the number of cell files, thus controlling the radial growth and thickness of the hypocotyl. Periclinal divisions result in derivative cells of different sizes or identities; for this reason, they are also referred to as “formative divisions” [39].

Present data show that these formative divisions occur in all treatments, including the untreated Controls. These cell divisions may be interpreted as a pericycle reaction to the seedling growth in the restricted environment of the in vitro culture, possibly for radially strengthening the hypocotyl base to sustain seedling growth. As in the controls, some proliferative response is also present in the presence of Cd, alone or combined with exogenous eBL. An increased number of cells in response to Cd is also observed in the pericycle of bean roots [37]. However, in Arabidopsis hypocotyls, the pollutant does not seem either able to increase per se the pericycle periclinal proliferation in comparison with the controls or to do it when combined with eBL. A strong enhancement of pericycle proliferation is instead caused by eBL alone, independently of the applied concentration. At similar low concentrations, but in combination with other exogenous phytohormones, brassinosteroids (BRs) stimulate cell proliferation in cultured parenchyma cells of *Helianthus tuberosus* [23] and Chinese cabbage mesophyll protoplasts [40]. However, in the absence of other phytohormones, BRs stimulate divisions during Arabidopsis root ground tissue development [41], and their signaling directs formative divisions in the root meristems [42]. Moreover, the fact that the induced proliferation is only periclinal clearly confirms an eBL-induced effect on microtubule remodeling [43], specific for phragmoplast orientation leading to periclinal cytokinesis.

Our data show that EXE formation is an indirect process because it has periclinal proliferation as a prerequisite. In fact, the EXE formation never occurs as direct transdifferentiation of the pericycle cells independently of the treatment.

An interesting result is that exogenous eBL at the very low concentration of 1 nM induces a reversal position of the metaxylem and protoxylem within the stele. In normal conditions, the stele of Arabidopsis hypocotyl shows an endarch metaxylem and exarch protoxylem [5]. However, there are cases of reversal in position, for example, those that occur in Arabidopsis roots under water limitation conditions [44] or in the hypocotyls of *Atshr* and *Atscr* mutants [4]. In our case, it seems improbable that the transcriptional switching from metaxylem to protoxylem is a stress response [16] because the event only occurs in the presence of 1nM eBL, with this concentration being more efficient alone than in combination with Cd. It is, thus, more probable that the BR action is related to the activity of the SHORTROOT (SHR)/SCARECROW (SCR) complex in controlling xylary identity. The GRAS transcription factor SHR is the activator of SCR, both involved in the xylogenic process by inducing the expression of microRNAs (miRNAs) miR165 and miR166. High levels of those miRNAs have been reported in relation to the expression of numerous xylem-specific target genes, indirectly controlling protoxylem identity; low levels have been found, instead, to control metaxylem identity [4,45,46]. However, the relationship between SHR and BRs is known, both in the form of positive interactions, with BRs inducing *SHR* expression, and negative interactions, with BRs antagonizing *SHR* expression, at least for Arabidopsis roots [41,47]. Based on the present result showing that wild-type seedlings treated with 1nM eBL exhibit the same effect of the hypocotyl in the *Atshr* null mutant, it is possible to hypothesize that the 1nM eBL concentration negatively affects *SHR* expression. This possibly activates transcription pathways alternative to the SHR/SCR or downstream to the complex, antagonizing miR165/miR166 activity. Our previous results showed that, in the hypocotyl of Arabidopsis *shr* and *scr* mutants, the stelar metaxylem/protoxylem reversal is associated with pericycle periclinal proliferation with EXE formation [4]. In accordance with the hypothesis of an antagonist relationship between BRs and SHR/SCR, present data show that 1 nM eBL causes the same two processes (xylem identity reversal in the stele and EXE formation in the pericycle derivatives) in the hypocotyl, and mainly when not combined with Cd.

Cadmium is known to cause precocious differentiation of primary tissues, including xylem formation in Arabidopsis roots [38] and protoxylem lignification in *Pinus sylvestris* [48], and to induce premature xylogenesis in barley roots [35]. However, in no case has Cd been reported to cause a metaxylem vs. protoxylem reversal in the stele, suggesting that the two events of xylary reversal and EXE formation are eBL-induced. Moreover, protoxylem is the xylary type prevalently formed in Cd-alone-induced pericycle proliferation (present results). The EXEs with protoxylem identity exhibit annular and spiral thickenings, whereas those with metaxylem identity are reticulate or pitted, in accordance with our previous results in the same organ under different hormonal conditions [5]. It is known that the metaxylem completes its differentiation later than the protoxylem [16], which occurs for both stelar and ectopic formation [5]. This means that in the presence of Cd (alone or combined with eBL), the ectopic protoxylem is formed more rapidly than the metaxylem, possibly as a pericycle response to increasing basal hypocotyl strength to the pollutant. Hydraulic conductance is an important function of the xylem in planta. To allow the long distant translocation of the raw sap, the xylem elements must be differentiated as a continuum. Present data show this is not the case for EXEs formed by the pericycle derivatives because they are differentiated randomly without continuity. For this reason, we hypothesize that the Cd-induced ectopic protoxylem formation is a way to fortify the hypocotyl as a mechanical barrier against the pollutant.

Interestingly, eBL highly enhances EXE formation when applied alone, affecting xylary cell identity, with the lower eBL concentrations favoring protoxylem formation and the highest one favoring metaxylem formation. The promotive role of BRs in xylogenesis in vitro is known [7,23], but the role of eBL for EXE formation in planta is unknown, as it is unknown that this compound affects xylary identity. We show that this is the case and that the prevalent expression of the xylary identity is concentration-dependent. It is not to be excluded that the pericycle derivatives produce EXEs as a stress-defense response at least at the highest exogenous eBL-alone concentration (1 µM), which induces metaxylem. Still, when combined with Cd, it causes a very reduced hypocotyl elongation and stunted seedling growth (present results). It is, however, of interest that the other two exogenous eBL concentrations result in a significant increase in EXE formation when combined with Cd without affecting the xylary identity, which remains of protoxylem as with Cd alone. Thus, these steroids may ameliorate seedling resistance to the pollutant by increasing EXE formation to form a mechanical defense barrier, but this depends on the exogenous/endogenous level. A short-term and long-term transcriptome analysis will be carried out in the presence of eBL and/or Cd to identify the genes involved in the xylogenic process.

In conclusion, specific levels of exogenous eBL enhance plant resistance to the pollutant Cd, positively affecting the morphogenic process of xylogenesis in planta. Present data open the way for a deeper insight into how this compound contributes to the overall plant growth program in Cd-polluted soils.

### Agricultural Perspectives

It is known that successful seedling development is the initial step to obtaining plants capable of resistance to soil contaminants. For this reason, seedling growth was monitored in numerous studies also using 24-epibrassinolide (eBL) [49]. We show that exogenous eBL treatments, at specific concentrations, promoted seedling growth, strengthening the hypocotyl base even in the presence of Cd pollution. The thickening of the hypocotyl base is an important feature for the negative gravitropism response of the shoot, which is essential for directing the growth of aerial organs away from the soil surface after germination and for keeping the correct plant posture above ground [50]. It has been recently shown that this role is induced by 24-epibrassinolide (eBL) [50], and it is histologically demonstrated for the first time that it occurs through an ectopic xylogenic response. Brassinosteroids are non-toxic and environmentally friendly compounds successfully used in the field, mainly as eBL and 28-homobrassinolide [51]. In this way, they are known to improve growth in rice and other crops in response to the same (Cd) or other pollutants [33,51]. Thus, based on its protective role against Cd stress, present data suggest that BR supplementation may be recommended for improving the yield and quality of crops, bringing a huge prospect in agriculture.

## 4. Materials and Methods

### 4.1. Plant Material and Growth Conditions

Seeds of *A. thaliana* (L.) Heynh ecotype Columbia (Col) were stratified and sterilized according to Della Rovere and co-workers [3] and then sown in vitro on a medium containing half-strength Murashige and Skoog [52] basal salt mixture, 0.5% sucrose and 0.8% agar at pH 5.8. To the control medium (Hormone-free, HF), either 24-epibrassinolide (eBL, Sigma-Aldrich-E1641, Saint Louis, MO, USA) at 1 nM, 10 nM, and 1 µM or 60 µM CdSO_4_ (Cd) [38], alone or combined with 1 nM, 10 nM, and 1 µM eBL, was added [34]. The Cd concentration was selected based on previous results showing its effects on morphogenesis when applied alone [38,53] or in combination with eBL in seedlings of the same plant [34]. Plates (12 cm × 12 cm; 10 seeds per plate) were placed in a vertical position and kept in a growth chamber at 22 ± 2 °C, 70% humidity. The plates were initially (6 h) exposed to white light (100 µEm^−2^ s ^−1^ intensity), then transferred to continuous darkness (9 days), and finally exposed to a 16 h light/8 h dark photoperiod for 7 days [34]. Ultrapure water (Milli-Q^®^, Merck, Germany) was used.

### 4.2. Histological and Autofluorescence Analysis

In total, thirty seedlings per experimental replicate and treatment were fixed in 70% ethanol (EtOH), dehydrated through a graded EtOH series, and embedded with Technovit 7100 embedding resin (Heraeus Kulzer, Germany). Then, the embedded seedlings were longitudinally sectioned at 8 μm intervals using the Microm HM 350 SV microtome (Microm, Walldorf, Germany) and stained with 0.05% toluidine blue according to Della Rovere et al. [3]. Sections were taken from the basal portion (5 mm in length) of the hypocotyl, according to Fattorini and co-workers [5], and observed with a Leica DMRB microscope. The images were acquired with an OPTIKA C-P20CC camera (Optika, Italy). The autofluorescence of the lignified cell walls was detected on sections observed under UV light using Axio Imager M2 (Zeiss, Germany) microscope equipped with an EX BP 359/371 and EM LP 397 filter set. The images were acquired with an Axiocam 512 (Zeiss, Germany) camera. The highly reduced growth of the seedlings treated with 1 µM eBL combined with 60 µM CdSO_4_ (Figure S1) prompted us to exclude this treatment from the histological analyses.

### 4.3. Measurements and Statistical Analysis

The hypocotyls of 30 seedlings per treatment were cut into three portions, and the basal portion was selected. The middle of this portion was used to measure the vascular system’s radial extension, including the de novo cells eventually formed by the pericycle periclinal divisions [5]. Measures were expressed as mean values ± standard errors (SEs), and the ectopic xylary elements (EXEs) present in an area of 150 × 150 µm^2^ were counted and quantified as mean numbers (±SEs). The protoxylem vs. metaxylem identity was expressed as a percentage of the total de novo formed EXEs. The xylary reversal (i.e., protoxylem formed in the place of metaxylem within the hypocotyl stele) was also quantified and expressed as a percentage of the seedlings showing the phenomenon. One-way analysis of variance (ANOVA, *p* < 0.05) was used to compare the effects of different treatments, and if ANOVA showed significant effects, Tukey’s post-test was applied (GraphPad Prism 6.0, GraphPad Software, Inc., La Jolla, CA, USA). All the experiments were repeated three times, with very similar results.

## Figures and Tables

**Table 1:** Quantification of ectopic xylary elements (EXEs) detected in the basal hypocotyl of *A. thaliana* (Col ecotype) seedlings cultured for 9 days under continuous darkness followed by 7 days under 16 h light/8 h darkness photoperiod either in a hormone-free medium (Control), or with 1 nM of 24-epibrassinolide (eBL), 10 nM eBL, 1 μM eBL, 60 µM CdSO_4_ (Cd), 60 μM Cd + 1 nM eBL, or 60 μM Cd + 10 nM eBL. Mean number [± standard error (SE)] of EXEs and percentage of ectopic protoxylem and metaxylem elements detected in an area of 150 × 150 µm^2^ in the middle portion of the basal hypocotyl. Letters indicate significant differences among values at least at *p* < 0.05. The same letter shows no significant difference. N = 30.

Treatment	Ectopic XEs Mean Number (± SE)	Ectopic Protoxylem (%)	Ectopic Metaxylem (%)
Control	0.51 ± 0.12 ^a^	80	20
1 nM eBL	1.4 ± 0.15 ^b^	80	20
10 nM eBL	1.55 ± 0.20 ^b^	50	50
1 µM eBL	1.82 ± 0.20 ^b^	35	65
Cd	0.62 ± 0.10 ^a^	80	20
Cd + 1 nM eBL	1.24 ± 0.10 ^b^	83	17
Cd + 10 nM eBL	1.26 ± 0.09 ^b^	80	20

## Data Availability

Not applicable.

## Supplementary Materials

The following supporting information can be downloaded at: https://www.mdpi.com/article/10.3390/plants11233278/s1, Figure S1: *A. thaliana* seedlings grown in vitro with Cd and eBL. Stereomicroscope images of *A. thaliana* seedlings (Col ecotype) cultured for 9 days under continuous darkness followed by 7 days under 16 h light/8 h darkness photoperiod on ½ MS medium plus 60 μM CdSO_4_ (Cd) + 1 nM epibrassinolide (eBL) (A), or 60 μM Cd + 10 nM eBL (B), or 60 μM Cd + 1 μM eBL (C). Bars = 5 mm.

## References

[1] Busse J.S., Evert R.F. (1999). Vascular Differentiation and Transition in the Seedling of *Arabidopsis thaliana* (Brassicaceae). Int. J. Plant Sci..

[2] Xiao W., Molina D., Wunderling A., Ripper D., Vermeer J.R.M., Ragni L. (2020). Pluripotent Pericycle Cells Trigger Different Growth Outputs by Integrating Developmental Cues into Distinct Regulatory Modules. Curr. Biol..

[3] Della Rovere F., Fattorini L., D’Angeli S., Veloccia A., Falasca G., Altamura M.M. (2013). Auxin and cytokinin control formation of the quiescent centre in the adventitious root apex of arabidopsis. Ann. Bot..

[4] Della Rovere F., Fattorini L., D’Angeli S., Veloccia A., Del Duca S., Cai G., Falasca G., Altamura M.M. (2015). Arabidopsis SHR and SCR transcription factors and AUX1 auxin influx carrier control the switch between adventitious rooting and xylogenesis *in planta* and in *in vitro* cultured thin cell layers. Ann. Bot..

[5] Fattorini L., Della Rovere F., Andreini E., Ronzan M., Falasca G., Altamura M.M. (2017). Indole-3-Butyric Acid Induces Ectopic Formation of Metaxylem in the Hypocotyl of *Arabidopsis thaliana* without Conversion into Indole-3-Acetic Acid and with a Positive Interaction with Ethylene. Int. J. Mol. Sci..

[6] Falasca G., Altamura M.M. (2003). Histological analysis of adventitious rooting in *Arabidopsis thaliana* (L.) Heynh seedlings. Plant Biosyst..

[7] Yamamoto R., Fujioka S., Demura T., Takatsuto S., Yoshida S., Fukuda H. (2001). Brassinosteroid Levels Increase Drastically Prior to Morphogenesis of Tracheary Elements. Plant Physiol..

[8] Fattorini L., Falasca G., Kevers C., Mainero Rocca L., Zadra C., Altamura M.M. (2009). Adventitious rooting is enhanced by methyl jasmonate in tobacco thin cell layers. Planta.

[9] Kondo Y., Fujita T., Sugiyama M., Fukuda H. (2015). A novel system for xylem cell differentiation in *Arabidopsis thaliana*. Mol. Plant..

[10] Kondo Y. (2022). Competitive action between Brassinosteroid and tracheary element differentiation inhibitory factor in controlling xylem cell differentiation. Plant Biotechnol..

[11] Iwasaki T., Shibaoka H. (1991). Brassinosteroids act as regulators of tracheary-element differentiation in isolated *Zinnia* mesophyll cells. Plant Cell Physiol..

[12] Fukuda H. (1997). Tracheary element differentiation. Plant Cell.

[13] Xu L. (2018). *De novo* root regeneration from leaf explants: Wounding, auxin, and cell fate transition. Curr. Opin. Plant Biol..

[14] Harju A.M., Venäläinen M., Laakso T., Saranpää P. (2009). Wounding response in xylem of Scots pine seedlings shows wide genetic variation and connection with the constitutive defence of heartwood. Tree Physiol..

[15] Miyashima S., Sebastian J., Lee J.-Y., Helariutta Y. (2013). Stem cell function during plant vascular development. EMBO J..

[16] Kubo M., Udagawa M., Nishikubo N., Horiguchi G., Yamaguchi M., Ito J., Mimura T., Fukuda H., Demura T. (2005). Transcription switches for protoxylem and metaxylem vessel formation. Genes Dev..

[17] Fattorini L., Hause B., Gutierrez L., Veloccia A., Della Rovere F., Piacentini D., Falasca G., Altamura M.M. (2018). Jasmonate promotes auxin-induced adventitious rooting in dark-grown *Arabidopsis thaliana* seedlings and stem thin cell layers by a cross-talk with ethylene signalling and a modulation of xylogenesis. BMC Plant Biol..

[18] Della Rovere F., Fattorini L., Ronzan M., Falasca G., Altamura M.M., Betti C. (2019). Jasmonic Acid Methyl Ester Induces Xylogenesis and Modulates Auxin-Induced Xylary Cell Identity with NO Involvement. Int. J. Mol. Sci..

[19] Lee J., Han S., Lee H.-Y., Jeong B., Heo T.-Y., Hyun T.K., Kim K., Je B.I., Lee H., Shim D. (2019). Brassinosteroids facilitate xylem differentiation and wood formation in tomato. Planta.

[20] Yamamoto R., Demura T., Fukuda H. (1997). Brassinosteroids Induce Entry into the Final Stage of Tracheary Element Differentiation in Cultured *Zinnia* cells. Plant Cell Physiol..

[21] Szekeres M., Németh K., Koncz-Kálmán Z., Mathur J., Kauschmann A., Altmann T., Rédei G.P., Nagy F., Schell J., Koncz C. (1996). Brassinosteroids Rescue the Deficiency of CYP90, a Cytochrome P450, Controlling Cell Elongation and De-etiolation in Arabidopsis. Cell.

[22] Choe S., Dilkes B.P., Gregory B.D., Ross A.S., Yuan H., Noguchi T., Fujioka S., Takatsuto S., Tanaka A., Yoshida S. (1999). The Arabidopsis *dwarf1* Mutant is Defective in the Conversion of 24-Methylenecholesterol to Campesterol in Brassinosteroid Biosynthesis. Plant Physiol..

[23] Clouse S.D., Zurek D., Cutler H.G., Yokota T., Adam G. (1991). Molecular Analysis of Brassinolide Action in Plant Growth and Development. Brassinosteroids: Chemistry, Bioactivity and Applications.

[24] Yamamoto R., Fujioka S., Iwamoto K., Demura T., Takatsuto S., Yoshida S., Fukuda H. (2007). Co-regulation of brassinosteroid biosynthesis-related genes during xylem cell differentiation. Plant Cell Physiol..

[25] Betti C., Della Rovere F., Piacentini D., Fattorini L., Falasca G., Altamura M.M. (2021). Jasmonates, Ethylene and Brassinosteroids Control Adventitious and Lateral Rooting as Stress Avoidance Responses to Heavy Metals and Metalloids. Biomolecules.

[26] Etchells J.P., Smit M.E., Gaudinier A., Williams C.J., Brady S.M. (2016). A brief history of the TDIF-PXY signalling module: Balancing meristem identity and differentiation during vascular development. New Phytol..

[27] Saito M., Kondo Y., Fukuda H. (2018). BES1 and BZR1 redundantly promote phloem and xylem differentiation. Plant Cell Physiol..

[28] Tanveer M., Shahzad B., Sharma A., Biju S., Bhardwaj R. (2018). 24-Epibrassinolide; an active brassinolide and its role in salt stress tolerance in plants: A review. Plant Physiol. Biochem..

[29] Fan F., Zhou Z., Qin H., Tan J., Ding G. (2021). Exogenous Brassinosteroid Facilitates Xylem Development in *Pinus massoniana* Seedlings. Int. J. Mol. Sci..

[30] Nolan T.M., Vukašinović N., Liu D., Russinova E., Yin Y. (2020). Brassinosteroids: Multidimensional Regulators of Plant Growth, Development, and Stress Responses. Plant Cell.

[31] Planas-Riverola A., Gupta A., Betegón-Putze I., Bosch N., Ibañes M., Caño-Delgado A.I. (2019). Brassinosteroid signaling in plant development and adaptation to stress. Development.

[32] Hasan S.A., Hayat S., Ahmad A. (2011). Brassinosteroids protect photosynthetic machinery against the cadmium induced oxidative stress in two tomato cultivars. Chemosphere.

[33] Sharma P., Kumar A., Bhardwaj R. (2016). Plant steroidal hormone epibrassinolide regulate—Heavy metal stress tolerance in *Oryza sativa* L. by modulating antioxidant defense expression. Environ. Exp. Bot..

[34] Della Rovere F., Piacentini D., Fattorini L., Girardi N., Bellanima D., Falasca G., Altamura M.M., Betti C. (2022). Brassinosteroids Mitigate Cadmium Effects in Arabidopsis Root System without Any Cooperation with Nitric Oxide. Int. J. Mol. Sci..

[35] Ďurčeková K., Huttová J., Mistrík I., Ollé M., Tamás L. (2007). Cadmium induces premature xylogenesis in barley roots. Plant Soil.

[36] Sabella E., Aprile A., Tenuzzo B.A., Carata E., Panzarini E., Luvisi A., De Bellis L., Vergine M. (2022). Effects of Cadmium on Root Morpho-Physiology of Durum Wheat. Front. Plant. Sci..

[37] Vázquez M.D., Poschenrieder C., Barceld Y. (1992). Cadmium in bean roots. New Phytol..

[38] Fattorini L., Ronzan M., Piacentini D., Della Rovere F., De Virgilio C., Sofo A., Altamura M.M., Falasca G. (2017). Cadmium and arsenic affect quiescent centre formation and maintenance in *Arabidopsis thaliana* post-embryonic roots disrupting auxin biosynthesis and transport. Environ. Exp. Bot..

[39] Smet W., De Rybel B. (2016). Genetic and hormonal control of vascular tissue proliferation. Curr. Opin. Plant Biol..

[40] Nakajima N., Shida A., Toyama S. (1996). Effects of brassinosteroid on cell division and colony formation of Chinese cabbage mesophyll protoplasts. Jpn. J. Crop. Sci..

[41] Tian Y., Zhao N., Wang M., Zhou W., Guo J., Han C., Zhou C., Wang W., Wu S., Tang W. (2022). Integrated regulation of periclinal cell division by transcriptional module of BZR1-SHR in *Arabidopsis* roots. New Phytol..

[42] Kang Y.-H., Breda A., Hardtke C.S. (2017). Brassinosteroid signaling directs formative cell divisions and protophloem differentiation in *Arabidopsis* root meristems. Development.

[43] Catterou M., Dubois F., Schaller H., Aubanelle L., Vilcot B., Sangwan-Norreel B.S., Sangwan R.S. (2001). Brassinosteroids, microtubules and cell elongation in *Arabidopsis thaliana*. I. Molecular, cellular and physiological characterization of the *Arabidopsis bul1* mutant, defective in the Δ^7^-sterol-C5-desaturation step leading to brassinosteroid biosynthesis. Planta.

[44] Ramachandran P., Augstein F., Nguyen V., Carlsbecker A. (2020). Coping With Water Limitation: Hormones That Modify Plant Root Xylem Development. Front. Plant. Sci..

[45] De Rybel B., Mähönen A.P., Helariutta Y., Weijers D. (2016). Plant vascular development: From early specification to differentiation. Nat. Rev. Mol. Cell Biol..

[46] Fan P., Aguilar E., Bradai M., Xue H., Wang H., Rosas-Diaz T., Tang W., Wolf S., Zhang H., Xu L. (2021). The receptor-like kinases BAM1 and BAM2 are required for root xylem patterning. Proc. Natl. Acad. Sci. USA.

[47] Li M., Li P., Wang C., Xu H., Wang M., Wang Y., Niu X., Xu M., Wang H., Qin Y. (2022). Brassinosteroid signaling restricts root lignification by antagonizing SHORT-ROOT function in Arabidopsis. Plant Physiol..

[48] Schützendübel A., Schwanz P., Teichmann T., Gross K., Langenfeld-Heyser R., Godbold D.L., Polle A. (2001). Cadmium-Induced Changes in Antioxidative Systems, Hydrogen Peroxide Content, and Differentiation in Scots Pine Roots. Plant Physiol..

[49] Soares T.F.S.N., dos Santos Dias D.C.F., Oliveira A.M.S., Ribeiro D.M., dos Santos Dias L.A. (2020). Exogenous brassinosteroids increase lead stress tolerance in seed germination and seedling growth of Brassica juncea L. Ecotoxicol. Environ. Saf..

[50] Somssich M., Vandenbussche F., Ivakov A., Funke N., Ruprecht C., Vissenberg K., VanDer Straeten D., Persson S., Suslov D. (2021). Brassinosteroids influence Arabidopsis hypocotyl graviresponses through changes in mannans and cellulose. Plant Cell Physiol..

[51] Liu J., Zhang D., Sun X., Ding T., Lei B., Zhang C. (2017). Structure-activity relationship of brassinosteroids and their agricultural practical usages. Steroids.

[52] Murashige T., Skoog F. (1962). A Revised Medium for Rapid Growth and Bio Assays with Tobacco Tissue Cultures. Physiol. Plant..

[53] Brunetti P., Zanella L., Proia A., De Paolis A., Falasca G., Altamura M.M., Sanità di Toppi L., Costantino P., Cardarelli M. (2011). Cadmium tolerance and phytochelatin content of *Arabidopsis* seedlings over-expressing the phytochelatin synthase gene *ATPCS1*. J. Exp. Bot..

