# Supplementary figures and images for: Convergence between Development and Stress: Ectopic Xylem Formation in Arabidopsis Hypocotyl in Response to 24-Epibrassinolide and Cadmium
Source: Plants (Basel). 2022 Nov 28;11(23):3278. doi: 10.3390/plants11233278 (PMC9739498; doi:10.3390/plants11233278)

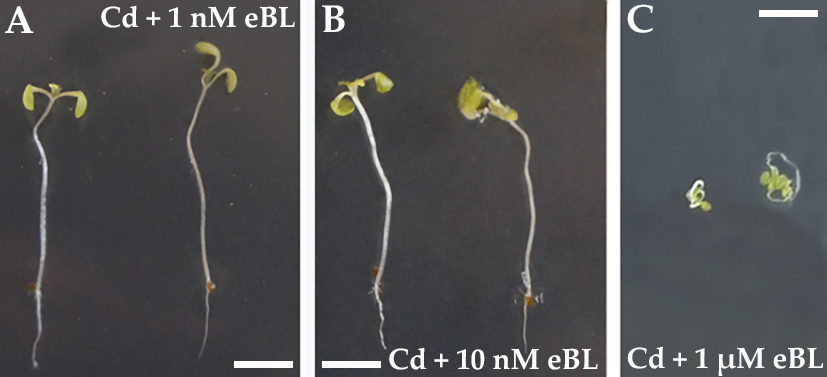

Supplement: Supplementary file 1 [file plants-11-03278-s001.zip › Figure S1.jpg]
